# Detection of recurrent cytogenetic aberrations in multiple myeloma: A comparison between MLPA and iFISH

**DOI:** 10.18632/oncotarget.5371

**Published:** 2015-09-17

**Authors:** Meirong Zang, Dehui Zou, Zhen Yu, Fei Li, Shuhua Yi, Xiaofei Ai, Xiaoqi Qin, Xiaoyan Feng, Wen Zhou, Yan Xu, Zengjun Li, Mu Hao, Weiwei Sui, Shuhui Deng, Chirag Acharya, Yaozhong Zhao, Kun Ru, Lugui Qiu, Gang An

**Affiliations:** ^1^ State Key Laboratory of Experimental Hematology, Institute of Hematology & Blood Diseases Hospital, Chinese Academy of Medical Science & Peking Union Medical College, Tianjin, China; ^2^ Cancer Research Institute, Key Laboratory of Carcinogenesis of Ministry of Health and Key Laboratory of Carcinogenesis and Cancer Invasion of Ministry of Education, Central South University, Changsha, Hunan, China; ^3^ LeBow Institute for Myeloma Therapeutics and Jerome Lipper Center for Multiple Myeloma Center, Dana-Farber Cancer Institute, Harvard Medical School, Boston, MA, USA; ^4^ Department of Hematology, The First Affiliated Hospital of Nanchang University, Jiangxi, Nanchang, China

**Keywords:** multiple myeloma, cytogenetic aberration, multiplex ligation-dependent probe amplification, interphase fluorescence *in situ* hybridization

## Abstract

Multiple myeloma (MM) is a genetically heterogeneous disease with diverse clinical characteristics and outcomes. Recently, multiplex ligation-dependent probe amplification (MLPA) has emerged as an effective and robust method for the detection of cytogenetic aberrations in MM patients. In the present study, MLPA analysis was applied to analyze cytogenetics of CD138 tumor cells of 59 MM samples, and its result was compared, retrospectively, with the interphase fluorescence *in situ* hybridization (iFISH) data. We firstly established the normal range of each of the 42 diagnostic probes using healthy donor samples. A total of 151 aberrations were detected in 59 patient samples, and 49/59 cases (83.1%) harbored at least one copy number variation. Overall, 0–7 aberrations were detected per case using MLPA, indicating the heterogeneity and complexity of MM cytogenetics. We showed the high efficiency of MLPA and the high congruency of the two methods to assess cytogenetic aberrations. Considering that MLPA analysis is not reliable when the aberration only exits in a small population of tumor cells, it is essential to use both MLPA and iFISH as complementary techniques for the diagnosis of MM.

## INTRODUCTION

Multiple myeloma (MM) is a terminally differentiated B cell neoplastic disorder, characterized by the presence of clonal proliferation of malignant plasma cells (PCs) in bone marrow and excessive monoclonal immunoglobulin associated with organ dysfunction [1]. Furthermore, MM is also a genetically heterogeneous disease, and whole-genome screening studies have shown that almost all MM patients harbor genetic abnormalities [2–4]. These genetic abnormalities can be caused by translocation of immunoglobulin heavy chain alleles (IgH) with various partner chromosome alleles, copy number variation (CNV) or acquired mutation [5]. As complex genome contributes to the initiation and progression of MM, cytogenetic aberration has emerged as the most important prognostic factor and is currently being used to predict the prognosis and make medical decisions for therapy [6].

Although conventional metaphase karyotyping offers a full view of chromosomes, alterations are only detected in 30% of MM cases due to the low mitotic activity of MM cells and the low resolution of the technique [7]. Interphase fluorescence *in situ* hybridization (iFISH) is able to overcome this shortcoming, and approximately 90% of abnormalities are reported when iFISH is applied for CD138 tumor cells [8]. Therefore, iFISH has emerged as the most viable and widely used approach to detect cytogenetic aberrations in MM. However, the problem remains since iFISH analysis is a laborious and time-consuming method with high cost, and is only capable to detect deletion or amplification of sequences larger than 20–50 kb. Furthermore, mutations cannot be detected by iFISH. In order to provide detailed evaluation of genomic complexity in MM, DNA microarray/comparative genome hybridization (CGH), next-generation sequencing (NGS), and gene expression profile (GEP) analysis have been carried out [8]. Although these novel techniques are more sensitive and amenable to higher throughput analysis, these assays are still difficult to be used in the routine clinical settings due to the cost and turnaround time.

Multiplex ligation-dependent probe amplification (MLPA) is a multiplex polymerase chain reaction (PCR) method for detecting CNVs up to 50 different genomic sequences simultaneously. MLPA probes are able to recognize target sequences with 50–100 nucleotides in length, which makes it possible to be applied for highly fragmented DNA, and the detection of small deletion encompassing only a single exon [9]. To date, hundreds of special panels have been developed and used successfully in the diagnosis of both benign and malignant diseases.

In this study, MLPA analysis was used to detect CNVs in MM using purified CD138 cells. The results were compared with iFISH data to determine the efficiency of MLPA. The combinatorial application of MLPA and iFISH in the routine diagnosis of MM was proposed.

## RESULTS

### Establishment of normal range for each individual MLPA probe

The X046-A1 MM probemix contained 53 MLPA probes (42 diagnostic probes and 11 reference probes) with amplified products between 122 and 499 nt, indicating that the PCR reaction efficiency was different among probes. As a result, it was inappropriate to use an arbitrary ratio range as the sole cutoff value for all probes, which was applied in most MLPA studies [10, 11]. Therefore, we established a normal range for each diagnostic probe (to be prognostically relevant) to improve the accuracy of MLPA analysis. A set of 10 DNA samples derived from peripheral blood of healthy donors were subjected to MLPA analysis. The range of each of the 42 diagnostic probes was calculated through two main steps: firstly, within every healthy donor sample, the peak area of a specific diagnostic probe was divided by the sum of the peak area of the 11 reference probes (intra-sample normalization); secondly, for each specific healthy donor sample, the peak value (derived from the first step) was divided by the average of the peak value (derived from the first step) of the other 9 healthy donors (inter-sample normalization). Finally, 10 values were reckoned for each diagnostic probe. There was a small standard deviation (SD) among the healthy donors for every probe. Consequently, “Mean ± 2SD” (95% CI, *p* = 0.05) and “Mean ± 3SD” (99% CI, *p* = 0.01) values for each individual probe were obtained, as listed in Table 1. For better evaluation of the results with a larger confidence interval (CI), the “Mean ± 3SD” reference range was used as the cutoff value for CNV determination in our study.

**Table 1:** Normal reference range established for each individual probe for MM MLPA

Target regions	Gene/Exon	Normal range (Mean ± 2SD; 95% CI, *p* = 0.05)	Normal range (Mean ± 3SD; 99% CI, *p* = 0.01)
1p32.3	FAF1, ex 4	0.85–1.17	0.77–1.25
1p32.3	CDKN2C, ex 3	0.83–1.19	0.75–1.28
1p32.3	PPAP2B, ex 1b	0.93–1.10	0.89–1.15
1p32.2	DAB1, ex 14	1.00–1.20	0.95–1.24
1p32.2	DAB1, ex 2	0.87–1.14	0.80–1.21
1p31.3	LEPR, ex 4	0.92–1.20	0.85–1.27
1p31.2	RPE65, ex 14	0.81–1.17	0.72–1.26
1p21.3	DPYD, ex 5	0.82–1.02	0.76–1.07
1p21.1	COL11A1, ex 45	0.78–1.07	0.71–1.14
1q21.3	CKS1B, ex 1b	0.81–1.15	0.72–1.24
1q21.3	CKS1B, ex 2	0.82–1.18	0.73–1.27
1q23.3	NUF2, ex 1b	0.83–1.13	0.75–1.21
1q23.3	NUF2, ex 14	0.90–1.14	0.84–1.20
1q23.3	RP11–541J2	0.80–1.16	0.71–1.25
1q23.3	RP11–541J2	0.91–1.16	0.85–1.22
1q23.3	RP11–541J2	0.82–1.05	0.76–1.10
1q23.3	RP11–541J2	0.76–1.15	0.67–1.24
1q23.3	RP11–480N10	0.82–1.12	0.75–1.20
1q23.3	PBX1, ex 9	0.87–1.17	0.80–1.25
5q31.3	PCDHA1, ex 1b	0.78–1.13	0.70–1.22
5q31.3	PCDHAC1, ex 1a	0.76–1.07	0.69–1.15
5q31.3	PCDHB2, ex 1	0.81–1.10	0.74–1.17
5q31.3	PCDHB10, ex 1	0.82–1.06	0.76–1.13
5q31.3	SLC25A2, ex 1	0.75–1.34	0.61–1.48
5q31.3	PCDHGA11, ex 1b	0.94–1.08	0.91–1.12
12p13.31	CD27, ex 3	0.79–1.08	0.72–1.15
12p13.31	VAMP1, ex 4b	0.78–1.06	0.71–1.13
12p13.31	NCAPD2, ex 2	0.84–1.17	0.76–1.25
12p13.31	NCAPD2, ex 32	0.74–1.18	0.63–1.29
12p13.31	CHD4, ex 40	0.80–1.13	0.72–1.21
12p13.31	CHD4, ex 2	0.67–1.15	0.55–1.27
13q14.2	RB1, ex 7	0.83–0.98	0.80–1.02
13q14.2	RB1, ex 27	0.75–1.18	0.65–1.28
13q14.3	DLEU2, ex 2	0.87–1.12	0.81–1.18
13q14.3	DLEU1, down	0.82–1.06	0.77–1.12
16q12.1	CYLD, ex 2	0.75–1.33	0.60–1.48
16q12.1	CYLD, ex 19	0.86–1.02	0.82–1.06
16q23.1	WWOX, ex 1a	0.82–1.04	0.76–1.09
16q23.1	WWOX, ex 8	0.93–1.08	0.89–1.12
17p13.1	TP53, ex 11	0.81–1.18	0.72–1.27
17p13.1	TP53, ex 8	0.85–1.05	0.80–1.10
17p13.1	TP53, ex 5	0.76–1.12	0.671.21

### Abnormalities detected by MLPA

**Figure 1:**
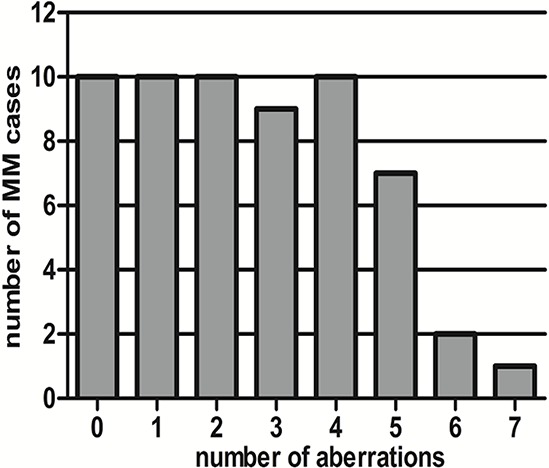
Number of aberrations present in each case detected by MLPA in this study

Sixty-four MM samples were subjected to MLPA analysis, and 59/64 (92.2%) cases passed the DNA quality control and were available for MLPA analysis. Overall, 151 aberrations were detected in 59 cases, and 49/59 cases (83.1%) harbored at least one CNV (Table 2). As shown in Figure 1, 0-7 aberrations were detected per case using MLPA, indicating the heterogeneity and complexity of MM cytogenetics.

**Table 2:** Summary of abnormalities detected by MLPA for per case

Sample number	Target region (number of affected probes/total number of probes)						
	Del (1p)	Amp (1q)	Amp (5q)	Del (12p)	Del (13q)	Del (16q)	Del (17p)
#1	1/9	4/10	5/6	ND	3/4	ND	ND
#2	ND	ND	ND	ND	4/4	ND	ND
#3	ND	8/10	ND	ND	ND	1/4	ND
#4	1/9	ND	1/6	ND	ND	ND	ND
#5	5/9	9/10	ND	1/6	1/4	2/4	ND
#6	ND	ND	ND	6/6	4/4	ND	ND
#7	ND	ND	ND	ND	ND	ND	ND
#8	3/9	ND	ND	6/6	4/4	ND	3/3
#9	1/9	10/10	5/6	ND	1/4	ND	3/3
#10	ND	ND	ND	ND	ND	ND	ND
#11	1/9	9/10	ND	ND	4/4	ND	ND
#12	1/9	ND	ND	ND	ND	ND	ND
#13	ND	ND	ND	ND	3/4	ND	ND
#14	1/9	9/10	4/6	ND	3/4	4/4	ND
#15	ND	10/10	ND	ND	4/4	ND	ND
#16	ND	ND	ND	ND	4/4	ND	ND
#17	7/9	10/10	5/6	ND	ND	ND	ND
#18	6/9	3/10*	2/6*	2/6	1/4	ND	ND
#19	ND	ND	ND	ND	4/4	ND	ND
#20	2/9	10/10	ND	1/6	4/4	4/4	3/3
#21	ND	ND	ND	ND	ND	ND	ND
#22	ND	10/10	ND	ND	ND	ND	ND
#23	ND	10/10	ND	ND	ND	ND	ND
#24	ND	ND	ND	ND	4/4	ND	ND
#25	7/9	8/10	ND	ND	4/4	ND	ND
#26	ND	ND	ND	ND	4/4	ND	3/3
#27	2/9	3/10	ND	ND	3/4	3/4	ND
#28	1/9	ND	ND	6/6	4/4	ND	ND
#29	2/9	2/10	1/6	1/6	4/4	ND	ND
#30	9/9	ND	ND	ND	4/4	4/4	3/3
#31	ND	ND	ND	1/6	ND	ND	ND
#32	8/9	4/10	ND	6/6	3/4	ND	ND
#33	3/9	4/10	ND	ND	3/4	ND	ND
#34	1/9	9/10	ND	ND	ND	ND	ND
#35	3/9	5/10	5/6	ND	3/4	1/4	ND
#36	ND	ND	ND	ND	ND	ND	ND
#37	5/9	4/10	5/6	1/6*	3/4	3/4	ND
#38	ND	ND	ND	ND	ND	ND	ND
#39	ND	10/10	6/6	ND	ND	ND	ND
#40	ND	ND	ND	ND	ND	ND	ND
#41	ND	2/10	5/6	1/6*	ND	ND	ND
#42	7/9	10/10	ND	ND	4/4	ND	ND
#43	ND	ND	ND	ND	ND	ND	ND
#44	ND	ND	ND	ND	ND	ND	ND
#45	ND	ND	ND	ND	ND	ND	ND
#46	ND	4/10	4/6	ND	ND	ND	ND
#47	2/9	3/10	6/6*	6/6	4/4	3/4	3/3
#48	ND	ND	ND	ND	1/4	2/4	ND
#49	ND	10/10	ND	ND	3/4	ND	ND
#50	3/9	10/10	ND	ND	3/4	ND	ND
#51	2/9	10/10	4/6	ND	3/4	3/4	ND
#52	2/9	8/10	5/6	ND	4/4	ND	ND
#53	1/9	6/10	ND	ND	1/4	1/4	ND
#54	ND	10/10	ND	ND	ND	ND	ND
#55	ND	ND	ND	1/6*	3/4	3/4	ND
#56	1/9	9/10	ND	ND	4/4	1/4	ND
#57	2/9	10/10	ND	6/6	ND	ND	3/3
#58	ND	ND	ND	ND	ND	ND	ND
#59	1/9	ND	1/6*	6/6	4/4	ND	ND

Del, deletion; Amp, amplification; ND, not detected.

Those with * are abnormalities different from the general situation, which would be discussed in detail in the results section.

**1p deletion** 1p deletion was detected in 30/59 cases (50.8%). There were 9 probes specially designed for short arm of chromosome 1, including 5 probes for 1p32, 2 probes for 1p31 and 2 probes for 1p21. Eleven cases only had one probe abnormality; while 8 cases displayed 1p deletion covering more than 3 probes. The DPYD gene at 1p21.3 was the most frequently affected gene (18/30), followed by PPAP2B at 1p32.2 (17/30), and COL11A1 at 1p21.1 (12/30).

**1q amplification** 1q amplification was detected in 32/59 cases (54.2%). Ten probes located in long arm of chromosome 1, including 2 probes for 1q21 and 8 probes for 1q23. Twenty-eight cases harbored large fragment amplification, which involved at least 4 probes, while only 4 cases showed amplification of less than 4 probes. All these 32 cases harbored 1q23 amplification, while 22 cases showed amplification of both 1q21 and 1q32. Contrary to 1p deletion, 1q amplification often represented large segment amplification, and 1q32 was more prone to amplification. In addition, 1q deletion was also detected in one case (#18) (* in Table 2). The involved probes were RP11–541J2 and RP11–480N10, which located at 1q23.3. However, the two probes spanned a gene-poor region, which rarely indicated prognostic impacts.

**5q amplification** 5q amplification was detected in 13/59 cases (22.0%). Six probes were included for the evaluation of 5q31.3. Eleven cases showed amplification covering more than 3 probes, while the other 2 cases showed amplification of only one single probe. However, 3 cases (#18, #47, #59) were detected to possess 5q deletion (* in Table 2). Sample #47 had deletion of all the six probes, while #18 and #59 had only two and one probe deletion, respectively.

**12p deletion** 12p deletion was detected in 12/59 cases (20.3%). Six probes hybridized to 12p13 were involved. Seven cases showed the loss of all six probes, indicating the large fragment deletion of 12p13. The other five cases showed only 1 or 2 probe abnormalities. The most frequently effected probes were CD27 (9/12), NCAPD2–2 (9/12) and CDH4–2 (9/12). However, we also found 3 cases (#37, #41, #55) with NCAPD2–2 amplification (* in Table 2).

**13q deletion** 13q deletion was detected in 36/59 cases (61.0%), which presented the most frequent aberration detected by MLPA. Two probes were designed for 13q14.2, while 2 probes were designed for 13q14.3. Thirty-one cases had the loss of large fragment, which involved at least 3 probes. The most frequently affected probe was RB1–7 (34/36), followed by DLEU1-down (33/36).

**16q deletion** 16q deletion was detected in 14/59 cases (23.7%). CYLD-2/-19 probes were particularly designed for 16q14.2, while WWOX-1/-8 probes were designed for 16q23.1. Ten cases showed the loss of at least 2 probes, and the most frequently affected probe was CYLD-19 (12/14), followed by WWOX-7 (10/14).

**17p deletion** 17p deletion was detected in 7/59 cases (11.9%). The 3 probes could target different exons of TP53, and were found lost in all 7 cases.

### Prognostic values of aberrations detected by MLPA

**Figure 2:**
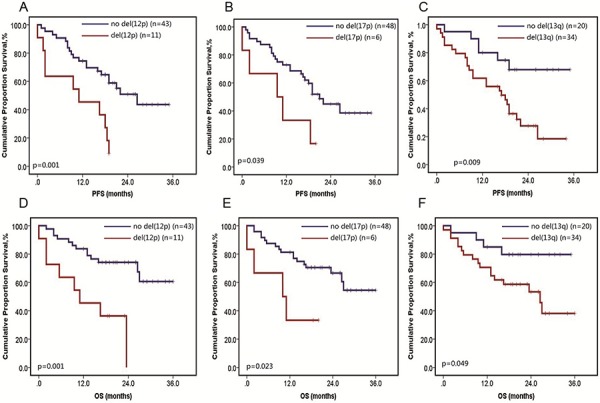
Survival analysis in MM patients harboring specific aberration detected by MLPA Progression free survival (PFS) of MM patients harboring del(12p) **A.** del(17p) **B.** del(13q) **C.** Overall survival (OS) of MM patients harboring del(12p) **D.** del(17p) **E.** del(13q) **F.**

These chromosomal aberrations detected by iFISH have already been found prognostically relevant. For instance, 5q31.3 amplification was reported to be a favorable prognostic factor of MM [2], while amplification of 1q, deletion of 1p, 12p, 13q, 16q and 17p were correlated with poor prognosis [5]. To explore the prognostic value of the same aberrations detected by MLPA, survival analysis was carried out in 54 MM samples with available follow up data. The results showed that patients with del(12p) had markedly shortened PFS (10.6 vs. 23.2 months, *p* = 0.001) and OS (12.8 vs. 27.8 months, *p* = 0.001) than those without del(12p) (Figure 2A and 2D). Meanwhile, patients with del(17p) were significantly worse than those without del(17p) with a median PFS of 10.2 vs. 21.8 months (*p* = 0.039) and a median OS of 10.5 vs. 26.3 months (*p* = 0.023) (Figure 2B, 2E). Moreover, 13q deletion could also cause shortened PFS of 16.9 vs. 27.6 months (*p* = 0.009) and OS of 29.8 vs. 22.2 months (*p* = 0.049) (Figure 2C and 2F). While the other aberrations, namely 1q amplification, 1p deletion and 16q deletion, failed to exhibit prognostic value separately. This may be caused by the small patient sample size and different treatment strategies. However, we found that three patients had at least five adverse lesions with a median OS of 2.5 months and a median PFS of 1.2 months, while patients with 1–4 adverse lesions had a median OS of 25.6 months and a median PFS of 20.4 months (*p* < 0.001).

### Comparison of MLPA and iFISH

To evaluate the performance of MLPA as a candidate diagnostic method for the identification of CNVs in MM, iFISH results of 59 cases were studied retrospectively and compared with MLPA results directly. The frequency of genetic lesions determined by iFISH and MLPA was listed in Table 3, and the combinatorial application of iFISH and MLPA revealed that 54/59 cases (91.5%) had at least one cytogenetic lesion.

**Table 3:** Frequency of genetic lesions determined by iFISH and MLPA (*N* = 59)

	iFISH % (*n* =)	MLPA % (*n* =)
**Del (1p)**	NA	50.8% (*n* = 30)
**Amp (1q)**	59.3% (*n* = 35)	54.2% (*n* = 32)
**Amp (5q)**	NA	22.0% (*n* = 13)
**Del (12p)**	NA	20.3% (*n* = 12)
**Del (13q)**	44.1% (*n* = 26)	61.0% (*n* = 36)
**IgH**	54.2% (*n* = 32)	NA
**Del (16q)**	NA	23.7% (*n* = 14)
**Del (17p)**	14.1% (*n* = 9)	11.9% (*n* = 7)
**Overall**	84.7% (*n* = 50)	83.1% (*n* = 49)

Del, deletion; Amp, amplification; NA, not analysis.

Three abnormalities, including 1q amplification, 13q deletion and 17p deletion were detected by both iFISH and MLPA. Out of 177 (3 × 59) comparisons, 156 results were concordant, and the whole consistency was 88.1%. The McNemar test was used for the comparison between iFISH and MLPA. As shown in Table 4, there were no significant differences between iFISH and MLPA results of 1q amplification and 17p deletion. The sensitivity and specificity of MLPA to determine 1q amplification were 91.4% and 100%; while the sensitivity and specificity of 17p deletion were 77.8% and 100%, respectively. The most obvious difference was observed in the determination of 13q deletion, with the sensitivity of 88.5% and the specificity of 60.6%.

**Table 4:** Sensitivities and specificities of MLPA compared with iFISH

		iFISH					
		Amp (1q)		Del (13q)		Del (17p)	
		+	−	+	−	+	−
**MLPA**	+	32	0	23	13	7	0
	−	3	24	3	20	2	50
**McNemar** **Test**		*p* = 0.250		*p* = 0.021		*p* = 0.500	

Del, deletion; Amp, amplification.

In 13 cases, 13q deletion was only detected by MLPA rather than iFISH. As displayed in Table 5, 4 cases (#5, #9, #18, #53) had only one probe deletion, which may lead to negative results of iFISH due to mutations or low resolution of the approach. Nevertheless, the remaining 9 cases with the abnormalities of at least 3 probes, spanning chromosomal regions including 13q14.2 and 13q14.3 were not detected by iFISH.

**Table 5:** Detailed discordances between MLPA and iFISH: aberrations only detected by MLPA but not iFISH (*n* = 13)

Sample number	iFISH mosaic (%)	Target regions (genes)
#5	2.5	13q14 (DLEU1)
#9	1.5	13q14 (RB1–7)
#14	3	13q14 (RB1–7, DLEU2, DLEU1)
#16	4	13q14 (RB1–7, RB1–27, DLEU2, DLEU1)
#18	5	13q14 (RB1–7)
#27	1	13q14 (RB1–7, DLEU2, DLEU1)
#29	0.5	13q14 (RB1–7, RB1–27, DLEU2, DLEU1)
#35	13.5	13q14 (RB1–7, DLEU2, DLEU1)
#37	4	13q14 (RB1–7, DLEU2, DLEU1)
#42	11	13q14 (RB1–7, RB1–27, DLEU2, DLEU1)
#51	3	13q14 (RB1–7, DLEU2, DLEU1)
#53	2.5	13q14 (DLEU1)
#55	5.5	13q14 (RB1–7, DLEU2, DLEU1)

It is worth noting that there were still 8 aberrations only detected by iFISH but not by MLPA, although MLPA showed a higher resolution. The discordant results were listed in Table 6. MLPA failed to detect the aberrations when the particular lesion was only present in a relatively small population. In detail, the clonal size of cases with 13q14 deletion detected by iFISH was 31%–100% (73.9 ± 22.9%), while cases not detected by MLPA (#58, #45, #57) possessed positive clones of 31%, 33% and 39%, respectively. For another example, the clonal size of 17p deletion detected by iFISH was 24.5%–97.5% (69.3 ± 22.4%), while cases not determined by MLPA (#6, #24) had positive clones of 24.5% and 46%. The similar situation occurred in 1q21 with the value 22.5%–100% (70.3 ± 25.3%). Cases #48, #12 and #40 had positive clones of 32%, 39% and 51%, respectively. Therefore, MLPA was prone to false negative results when the particular lesions occurred in a relatively small population.

**Table 6:** Detailed discordances between MLPA and iFISH: aberrations only detected by iFISH but not by MLPA (*n* = 8)

Sample number	iFISH mosaic (%)	Target regions (genes)
#12	39	1q21 (CKS1B)
#40	51	1q21 (CKS1B)
#48	32	1q21 (CKS1B)
#45	33	13q14 (RB1)
#57	39	13q14 (RB1)
#58	31	13q14 (RB1)
#6	24.5	17p13 (TP53)
#24	46	17p13 (TP53)

## DISCUSSION

Although MM patients present common histological and morphological diagnosis, MM displays enormous cytogenetic complexity and marked variations in clinical characteristics and outcomes. Thereby, risk stratification and prognosis assessment based on cytogenetic changes are important for treatment of MM. It has been well established that del(17p), t(4;14) and t(14;16) confer high risk of MM [3, 12]. However, some other certain cytogenetic changes also reveal significant impacts on outcomes. Several research groups have demonstrated that amp(1q), del(1p), del(12p) and del(16q) are adverse prognostic markers of MM [13–18]. Recent studies have also found that del(6q) worsen the prognosis of patients with del(17p) [19]. Therefore, it is highly necessary to reveal all prognostically relevant lesions in MM cells. Nevertheless, it is difficult for iFISH to screen all lesions simultaneously due to the high cost and technique limitation.

MLPA can analyze up to 50 CNVs in a single PCR reaction. Owing to the high throughput capability of the technique, MLPA analysis kit can be updated rapidly according to the latest research progress in cytogenetic study. For example, compared with SALSA P425-A1 kit, P425-B1 kit also contains probes related to amplification of chromosome 9 and 15 [10]. As a result, MLPA has emerged as an effective and robust method for the diagnosis of many diseases. In this study, we used MLPA to scan CNVs that are closely associated with MM prognosis based on published literature. However, we did not use a universal cutoff value for all probes. Instead, we established a normal range for each individual probe using a panel of 10 healthy donor samples. This will eliminate the difference in PCR efficiency of each probe and increase the accuracy of the experiments. To the best of our knowledge, this is the first report on the use of a specific range for each probe in MM investigation using MLPA. Donat et al. have used the same MLPA panel to detect CNVs in 81 MM patients [11]. The frequencies of 1q, 5q amplification and 12p, 13q, 16q, 17p deletion in our cohort are consistent with previous reports. The highest inconsistency occurred in the determination of 1p deletion, which was 35% in our study and 50.8% in Donat's study. The possible reason for this discrepancy is the different cutoff value. Values below or equal to 0.75 were considered as loss in Donat's study, while the cutoff value was higher than 0.75 in 6/9 probes in our study (Table 1). When the same cutoff value in Donat's study was applied, the 1p deletion rate was 40.0% in our cohort. Therefore, the “custom built” cutoff value improves the sensitivity of 1p detection by MLPA. However, whether the increased sensitivity is accompanied by false positive rate still needs investigation with a larger sample size and by other techniques.

MLPA results were compared with iFISH data because iFISH is the most commonly used method. Although there is a great concordance between iFISH and MLPA, some discrepancies still exist. In our study, the highest discrepancy exists in 13q deletion, with especially low specificity of 60.6%. The discrepancy may result from different probes used in the two techniques. In our study, del(13q) abnormality detected by iFISH was analyzed with the probe specific for 13q14.1–13q14.3 locus (LSI RB-1), which is approximately 220 kb and contains sequences targeting the entire RB1 gene. While in MLPA analysis, the two probes located in 13q14.2 only target exon 7 and exon 27 of RB-1 gene. The different resolution of the two techniques may be a reason of the differences. Moreover, the genes DLEU-1 and DLEU-2, targeting 13q14.3 in MLPA, located about 2.0 Mb far from the probe LSI RB-1 [20]. So some MM patients with 13q14.3 deletion may not be detected by iFISH in our study.

To conclude, discrepancies between the two methods may be related to three major reasons: different resolution, point mutation and subclone. Firstly, iFISH analysis is only able to detect deletion or amplification of sequences larger than 20–50 kb, while MLPA can recognize sequences of 50–100 nt in length. Consequently, the application by MLPA can achieve the detection of highly fragmented cryptic lesion. Secondly, mutation or polymorphism in the sequence detected by a probe can also result in reduction of the relative peak area in MLPA analysis, which cannot be picked up by iFISH. Usually, several different probes for a specific gene or different genes in the same chromosomal region are used in a MLPA kit. It is much more reliable when several related probes show the same aberration simultaneously. However, it is often ambiguous when only one single probe is used for the detection of abnormality. We cannot discriminate mutation or deletion without using other methods. Thirdly, with the development of novel technologies, such as CGH and NGS, clonal heterogeneity and clonal evolution have emerged as critical concepts in the field of oncology, especially in the pathogenesis of MM [21–23]. It is well known that genetic hits in MM are acquired not only in a linear fashion but also through a branching pattern [24]. In another word, subclones carrying different aberrations and distinct phenotypes may coexist in a single MM patient. However, MLPA analysis of tumor samples provides information of the total cells using their extracted DNA. If specific genetic aberration exists only in a very small subclone, it is likely not going to be detected. On the contrary, aberration present in each single tumor cell can be detected by iFISH. This is an important difference between these two methods. It is reported that MLPA will be effective when the abnormality is present in more than 30% of analyzed cells [11, 25]. But in our study, the threshold was more likely to be around 50%. The disparity may be caused by the differences in the contents of tumor cells. In Donat's study, tumor cell enrichment was applied only when the proportion of PCs was less than 20%–25%, while our MLPA analysis was conducted solely on purified CD138 cells. Therefore, we can conclude that the content of tumor cells has a great impact on the sensitivity of MLPA analysis, especially for MM diagnosis. Our previous study has demonstrated that prognostic values can be observed when patients harbor a small clone of 13q deletion or 1q21 amplification, such as 10% for 13q deletion and 20% for 1q21 amplification [13]. As a result, MLPA analysis is not reliable when the aberration only exits in a very small population of tumor cells.

Furthermore, MLPA failed to detect IgH translocation, which accounts for 40%–50% of primary cytogenetic events in MM and strongly influences disease phenotype and prognosis. This shortage of MLPA makes iFISH irreplaceable. Taken together, MLPA can detect CNVs in a high throughput fashion with higher resolution, while iFISH can detect both balanced and unbalanced rearrangements and are more reliable to detect aberrations existing in a small subclone. MLPA and iFISH analysis are mutually complementary for the detection of cytogenetic aberrations of myeloma.

## MATERIALS AND METHODS

### Patients and sample preparation

Bone marrow samples from 64 newly diagnosed MM patients were obtained at The Institute of Hematology and Blood Disease Hospital from August 2011 to March 2013. The male to female ratio was 40/24, and the median age of the patients was 58 years old (range: 25–75 years old) with the median follow-up time of 17 months (range: 0–36 months) from the time point of sample collection. Ten peripheral blood samples from healthy donors were also collected. All patients and healthy donors signed an informed consent form approved by institutional local ethics committee.

Mononuclear cells (MNCs) from MM patients and healthy donors were isolated by gradient density centrifugation (Ficoll-Hypaque; Eurobio, Les Ulis, France). Malignant PCs were purified from MM samples using the Miltenyi technology (anti-CD138-coated magnetic beads) as described previously to ensure plasma cell purity of higher than 90% [12].

### Interphase FISH analysis

Interphase FISH analysis was used to detect cytogenetic aberrations in purified PCs of MM. In detail, Del (13q) abnormality was analyzed with the probe specific for the 13q14 locus (LSI RB-1, Abbott Laboratories). Del (17p13) was assessed using a probe specific for the 17p13.1 locus (LSI p53, Abbott Laboratories). In order to detect the amplification of 1q21, we used 1q21 (CKS1B) probe (GP Medical Technologies, Beijing). The LSI IGH/FGFR3 dual-color probe (Abbott Laboratories), LSI IGH/CCND1 XT probe (Abbott Laboratories) and IGH/MAF DF probe (Abbott Laboratories) were used to detect t(4;14), t(11;14), and t(14;16), respectively.

A total of 200 interphase nuclei were analyzed. Cut-off values recommended by the European Myeloma Network (EMN) were used. For the deletion and numerical aberration, the cut-off level was set at 20%; for translocation in IgH locus as well as other translocation, the cut-off level was set at 10% [26].

### MLPA analysis

DNA was extracted using QIAamp DNA mini kit (Qiagen, Hilden, Germany), and subjected to MLPA analysis using SALSA kit X046-A1 that was renamed as SALSA P425-A1 (kindly donated by MRC Holland, Amsterdam, Netherlands). The probe mix contained 42 probes for the following chromosomal regions such as 1p32-p21, 1q21.3, 1q23.3, 5q31.3, 12p13.31, 13q14, 16q12, 16q23 and 17p13. These probes could predict prognostic relevance in MM. In addition, 11 reference probes were included in this probe mix for detecting 11 different chromosomal locations with relative quietness in MM. MLPA reactions were performed according to the manufacturer's instructions. Finally, the PCR products were analyzed using an ABI 3130 capillary sequencer (Applied Biosystems, Foster City, CA, USA) and http://Coffalyser.Net software (MRC Holland, Amsterdam, Netherlands) (Supplementary Figure).

### Statistical analysis

The McNemar test was used for the comparison between MLPA and iFISH for the aberration detection. Survival curves were plotted by the Kaplan-Meier method, and the difference was assessed by log-rank test. Progression free survival (PFS) was calculated from the initiation of therapy to the date of death, progression or last follow-up. Overall survival (OS) was measured from the initiation of treatment to the date of death or last follow-up according to the International uniform response criteria [27]. The statistically significant difference was considered at *p* < 0.05.

## SUPPLEMENTARY FIGURE


