# Supplementary material for: Detection of recurrent cytogenetic aberrations in multiple myeloma: A comparison between MLPA and iFISH
Source: Oncotarget. 2015 Sep 17;6(33):34276–87. doi: 10.18632/oncotarget.5371 (PMC4741451; doi:10.18632/oncotarget.5371)
Supplement: Supplementary file 1 [file oncotarget-06-34276-s001.pdf]

SUPPLEMENTARY FIGURE

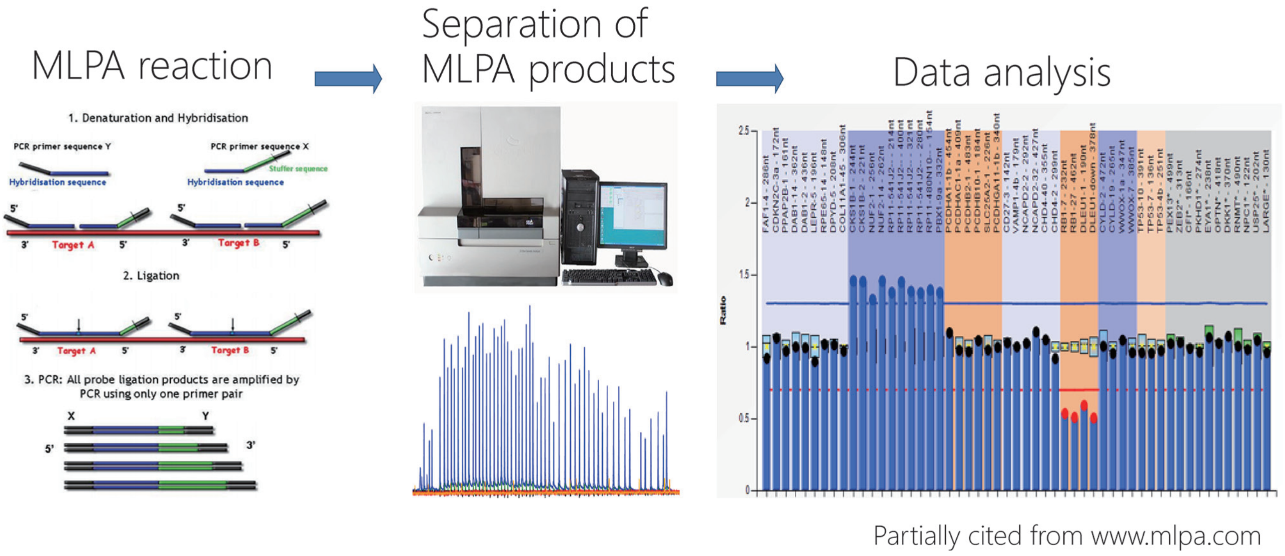

Supplementary Figure S1: General overview of the MLPA principle and procedural steps.
